# Supplementary material for: Sulfatase modifying factors control the timing of zebrafish convergence and extension morphogenesis
Source: Nat Commun. 2026 Mar 31;17:4632. doi: 10.1038/s41467-026-70804-6 (PMC13199485; doi:10.1038/s41467-026-70804-6)
Supplement: Supplementary file 1 — Supplementary Information [file 41467_2026_70804_MOESM1_ESM.pdf]

Supplementary materials

Supplementary Figures (S1-S9) and legends

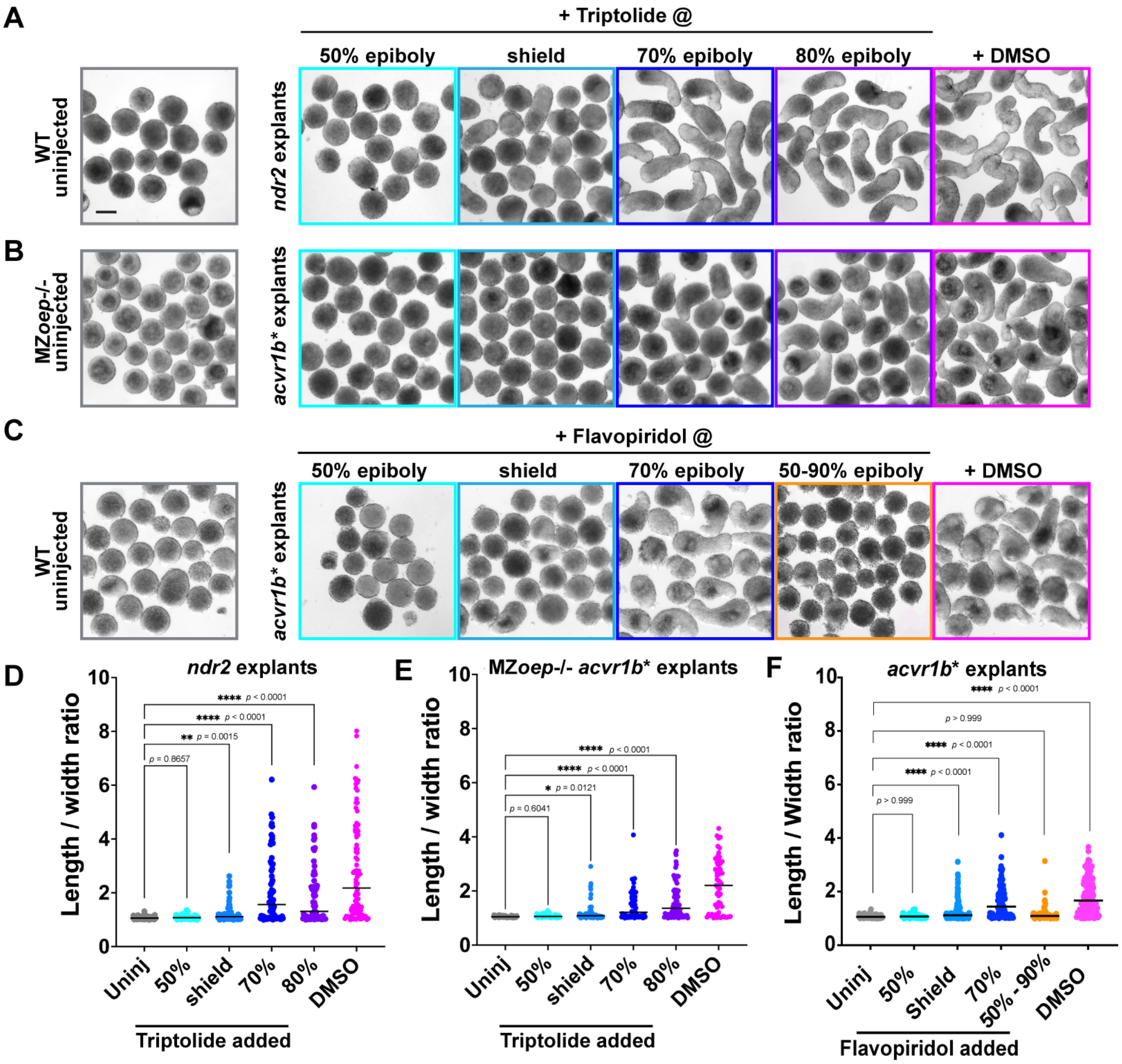

Supplementary Fig. 1. *Ex vivo* convergence & extension requires new gene expression at gastrulation onset independent of Nodal signaling dynamics. **(A, B)** Representative images of WT uninjected and *ndr2* explants (A), or MZ*oep*-/- uninjected and *acvr1b** explants (B) at 12 hpf (equivalent of 4-somite stage) after treatment with triptolide at the indicated stages or with DMSO at 50% epiboly. Scale bar = 200 μm. **(C)** Representative images of WT uninjected and *acvr1b** explants at 12 hpf (equivalent of 4-somite stage) after treatment with reversible transcription inhibitor Flavopiridol at the indicated stages or with DMSO at 50% epiboly. **(D-F)** Length/width ratios of explants shown in (A, B, and C), respectively. Each dot represents a single explant from three independent trials, black bars are median values; p<0.0001, Mann-Whitney test. Source data are provided as a supplementary Source Data file.

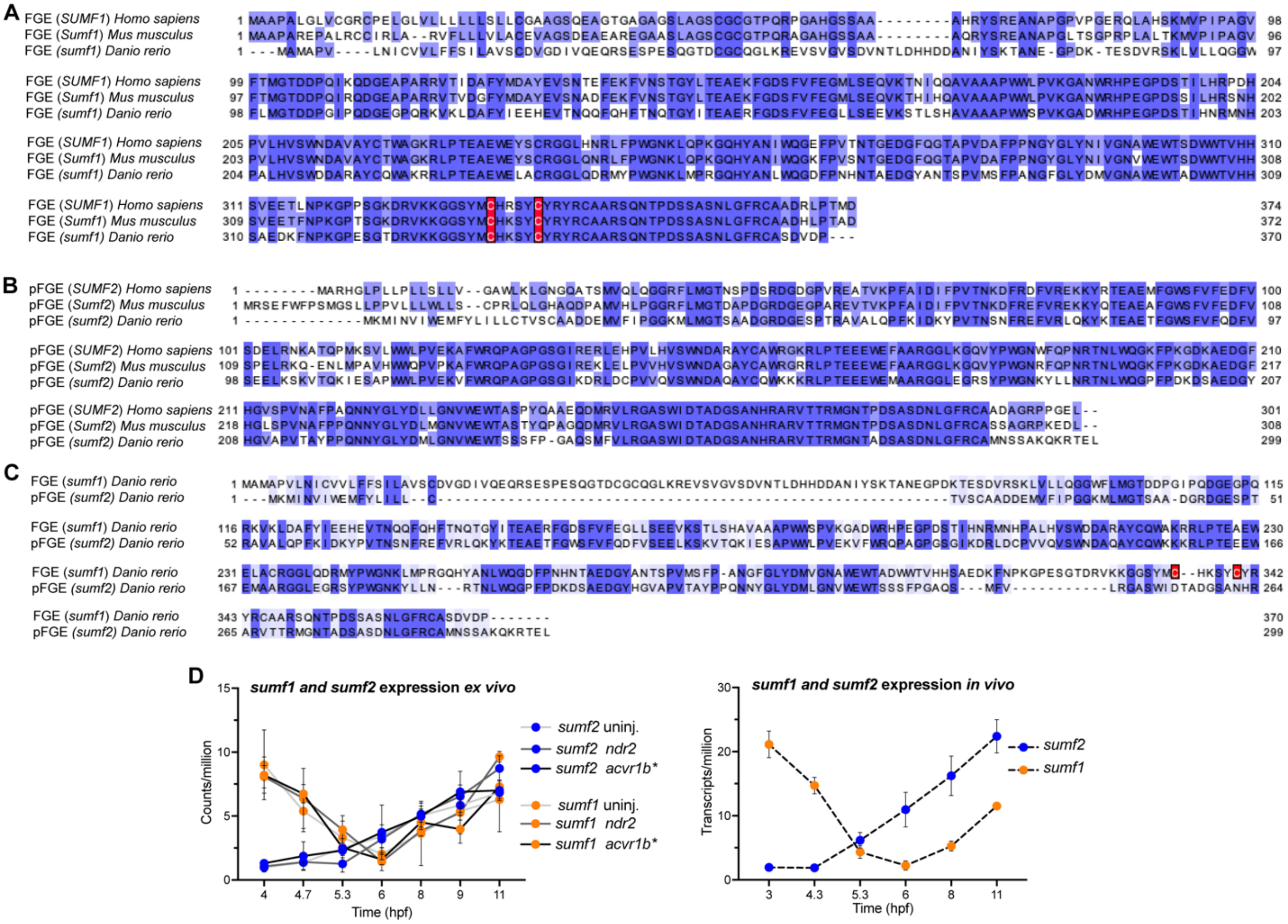

Supplementary Fig. 2. *sumf1* and *sumf2*-encoded protein sequence alignment and transcript expression during gastrulation. (A-C) Amino acid sequence alignments. (A) Alignment of FGE *(SUMF1)* homologs from human (*Homo sapiens*), mouse (*Mus musculus*), and zebrafish (*Danio rerio*). (B) Alignment of pFGE (*SUMF2*) homologs from the same species. (C) Pairwise alignment of zebrafish FGE (*sumf1)* and pFGE (*sumf2)* showing 46% amino acid identity and 62% similarity. Identical residues are highlighted in purple and conserved substitutions are highlighted in lilac. The two cysteine residues essential for FGE enzymatic activity are highlighted in red. **(D)** Expression levels of *sumf1* and *sumf2* in uninjected (light grey lines), *ndr2* (grey lines) and *acvr1b** (black lines) explants (left), and embryos (dashed lines, right) over time. Dots represent the mean of three (for explants) or five (for embryos) bulk RNA-seq replicates and error bars represent standard deviation. Source data are provided as a supplementary Source Data file.

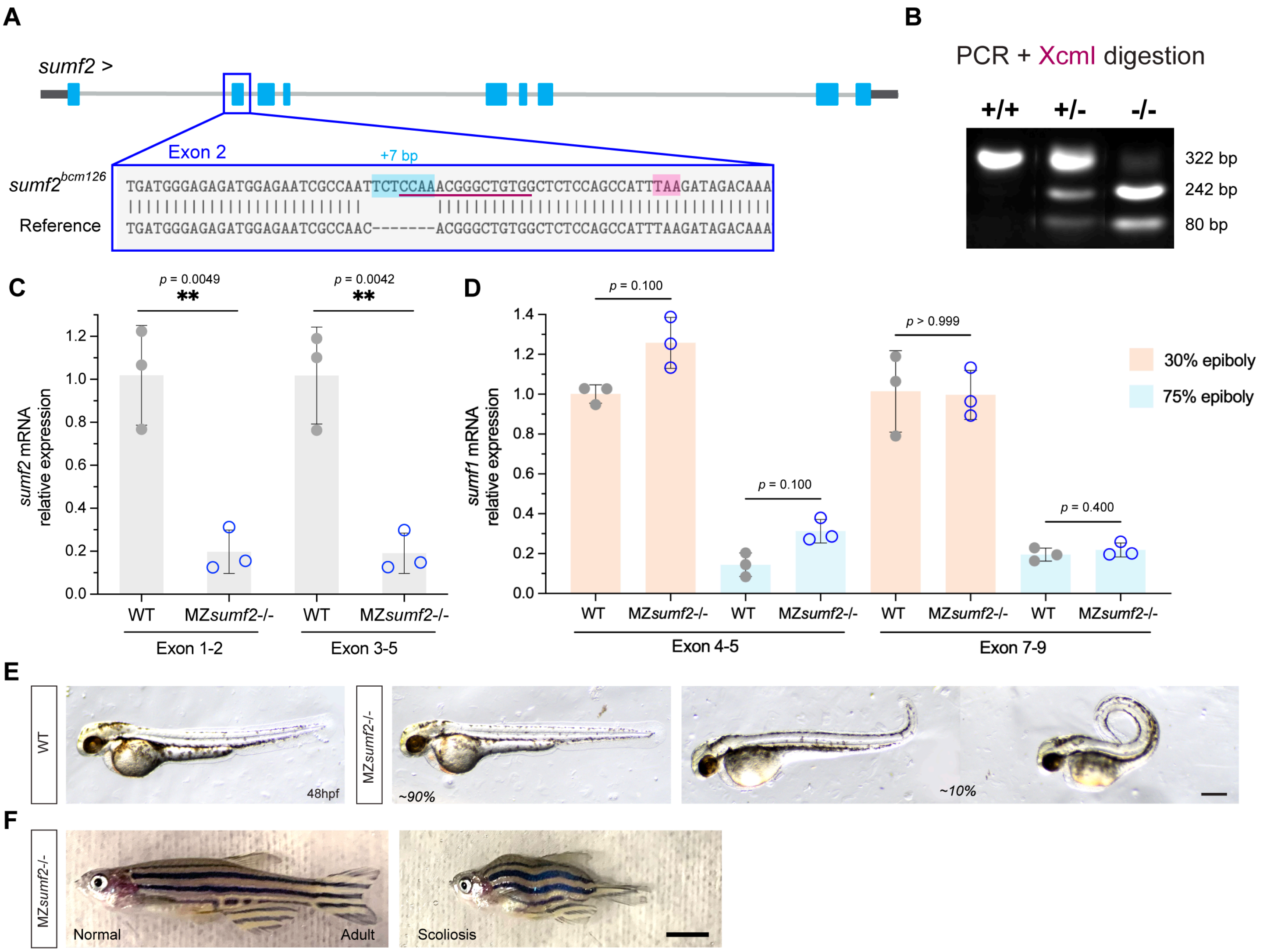

Supplementary Fig. 3. Characterization and genotyping of new *sumf2* loss-of-function allele. (A) Diagram of CRISPR-induced 7 bp insertion (cyan shading) in exon 2 of *sumf2* (*sumf2*^{bcm126}), resulting in a premature stop codon (magenta shading). **(B)** The *sumf2*^{bcm126} allele creates an XcmI restriction site (purple underline in (A)), enabling genotyping by digestion of a 322 bp PCR-amplified fragment, which yields 242 bp and 80 bp products in heterozygous (+/−), and homozygous (−/−) but not WT (+/+) samples. **(C)** Relative expression of *sumf2* transcripts in WT and MZ*sumf2*-/- larvae, measured by RT-qPCR using primers spanning *sumf2* exon 1–2 and exon 3–5. **(D)** Relative expression of *sumf1* transcripts in WT and MZ*sumf2*-/- at early (30% epiboly, 4.66 hpf) and mid (75% epiboly, 8 hpf) gastrulation, measured by RT-qPCR using primers spanning *sumf1* exon 4-5 and exon 7-9. Means and standard deviation are indicated. Each dot represents one of three independent clutches; ** p<0.01, Mann-Whitney test. **(E)** Representative images of WT and MZ*sumf2* -/- larvae at 48 hpf. Approximately 90% of MZ*sumf2*-/- larvae developed normally, while ~10% displayed varying degrees of tail-curled-up phenotypes. Anterior is to the left; scale bar = 200 µm. **(F)** Representative images of MZ*sumf2*-/- adult fish showing *Normal* and *Scoliosis* phenotypes. Scale bar = 500 µm. Source data are provided as a supplementary Source Data file.

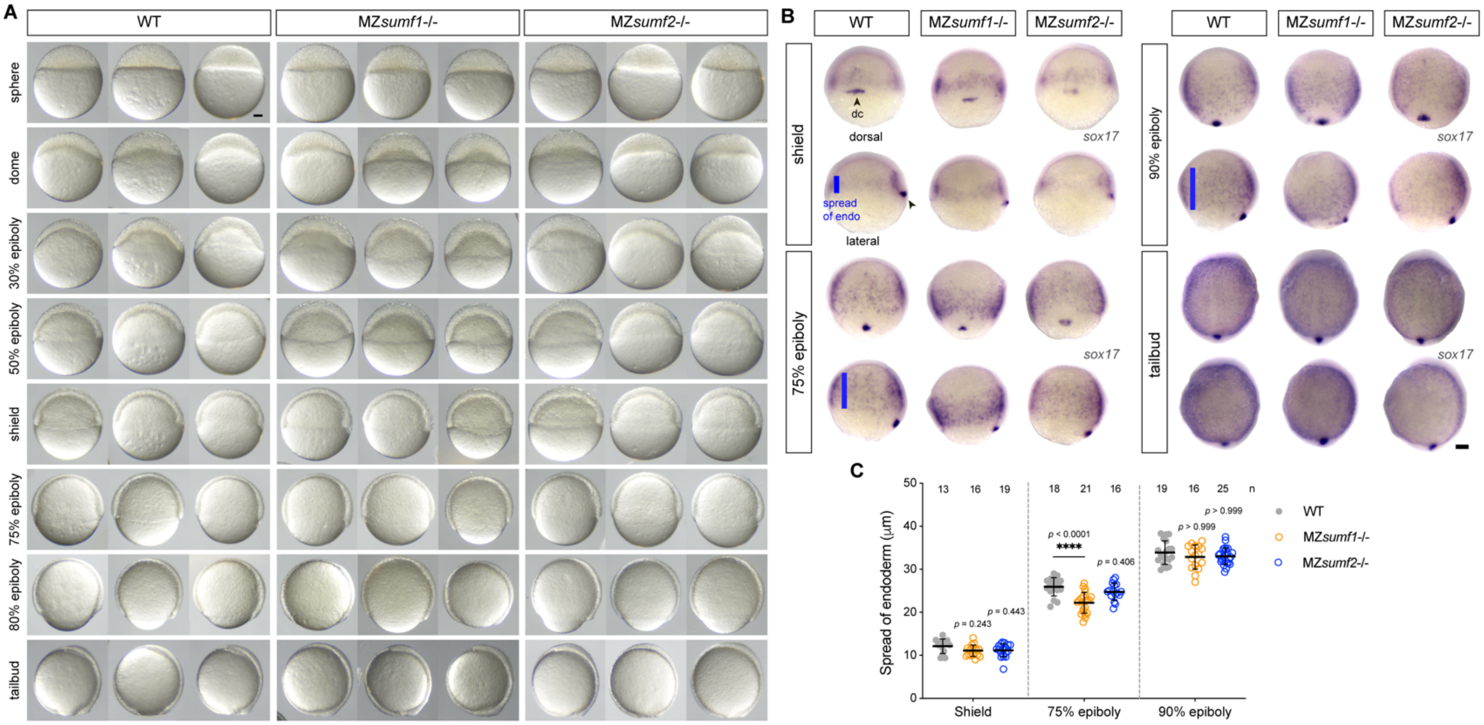

Supplementary Fig. 4. Epiboly and ingression morphogenesis are not substantially affected by loss of *sumf1* or *sumf2* function. (A) Representative images of live gastrulae of the indicated genotypes throughout gastrulation. Scale bar = 100 µm. **(B)** Representative images of whole mount in situ hybridization (WISH) for *sox17* (endoderm) in shield, (6 hpf), 75% epiboly (8 hpf), 90% epiboly (9 hpf) and tailbud (10 hpf) stage embryos of the indicated genotypes. Animal/anterior is up in all images, dorsal views are shown on top and lateral views on bottom (dorsal to the right). *dfc*: dorsal forerunner cells; blue bars represent endoderm spreading. Scale bar = 100 µm. Imaging and fixation of the embryos in A and B were conducted when age-matched WT embryos reached the indicated stages. **(C)** Endoderm spreading of embryos depicted in (B). Each dot represents a single embryo, n: number of embryos. Means and standard deviation are indicated; **** p<0.0001 compared to WT control group by Kruskal–Wallis and Dunn's multiple comparisons tests. Source data are provided as a supplementary Source Data file.

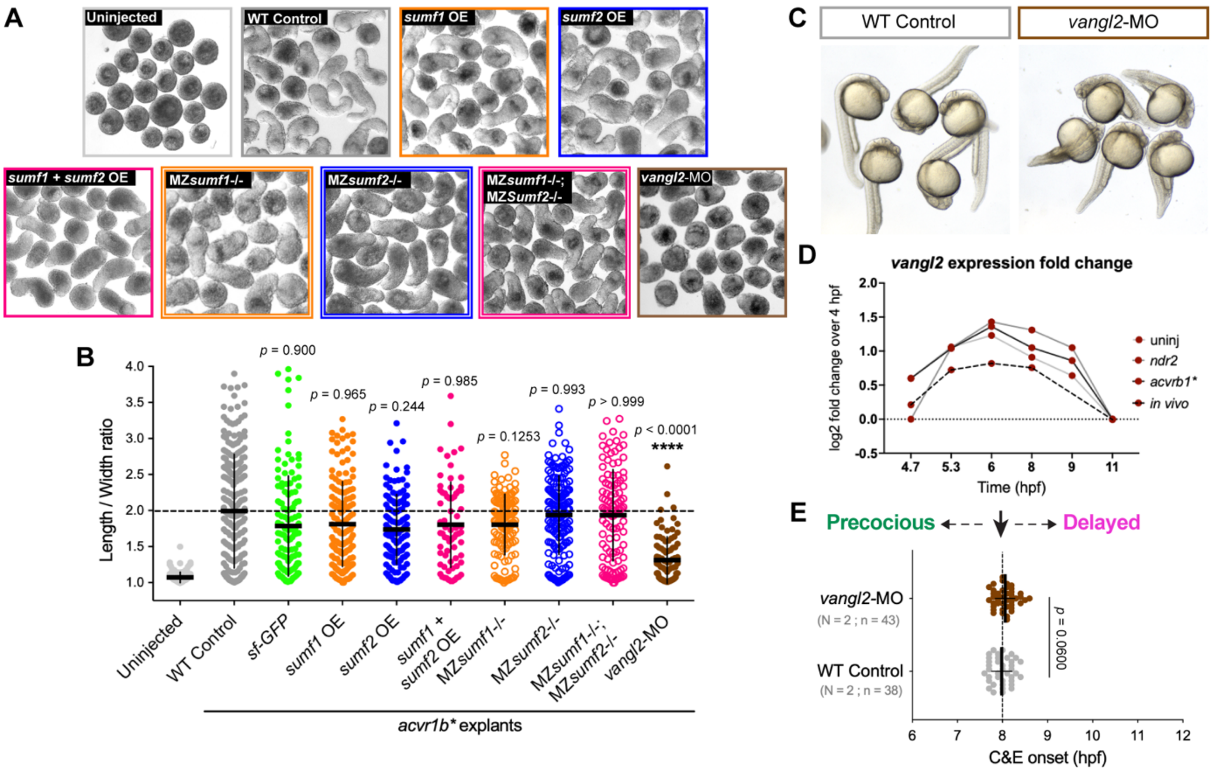

Supplementary Fig. 5. General C&E defects are distinct from altered C&E timing in explants. (A) Representative images of uninjected and *acvr1b** explants from the indicated experimental conditions. **(B)** Length/width ratios of explants at the equivalent of 4-somite stage (12 hpf) shown in (A). Each dot represents a single explant from three independent trials. Means and standard deviation are indicated. **** p<0.0001 compared with WT control group by Kruskal–Wallis and Dunn's multiple comparisons tests. **(C)** Representative images of WT Control and *vangl2* morpholino (MO)-injected embryos at 24 hpf. Note *vangl2*-MO larvae exhibit a shortened and widened body axis and cyclopia, typical of C&E defects. **(D)** Fold-change expression of *vangl2* transcripts over 4 hpf in explants (solid lines) and embryos (dashed lines) over time. **(E)** Onset of extension in WT Control and *vangl2*-MO *acvr1b** explants. Each dot represents a single explant. Means and standard deviation are indicated, N: number of independent experiments, n: number of explants, p>0.5 by Mann-Whitney test. Source data are provided as a supplementary Source Data file.

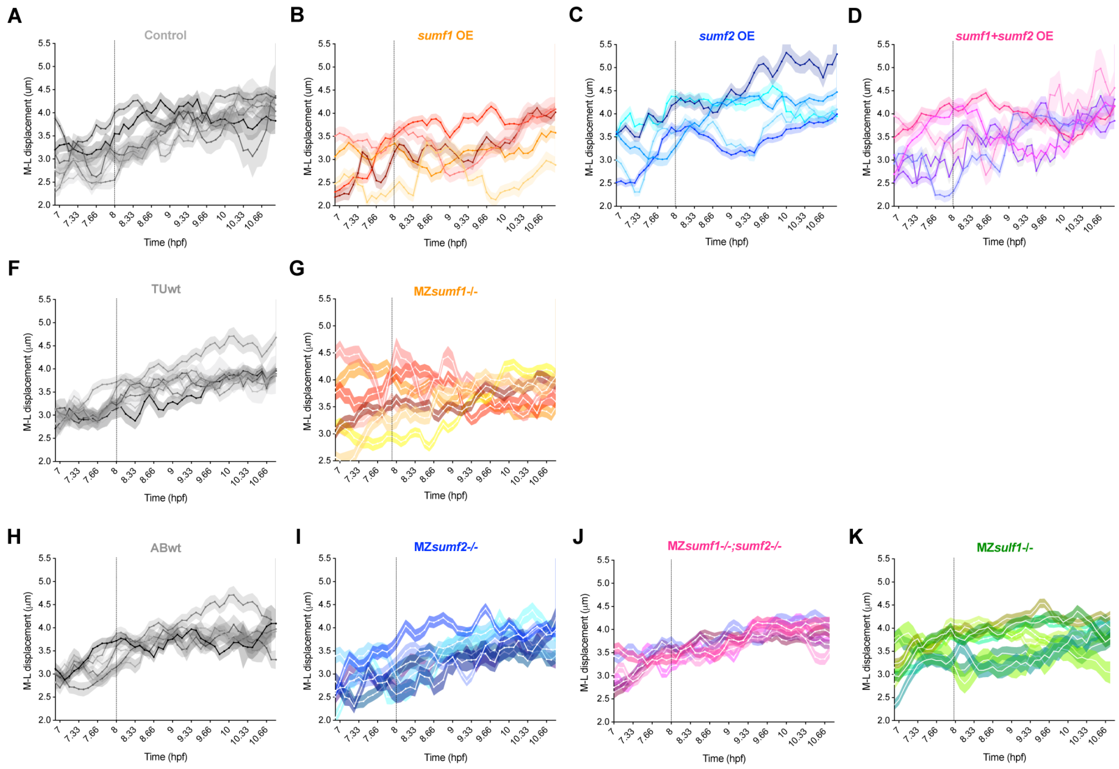

Supplementary Fig. 6. Mediolateral cell displacement over time within individual embryos. (A) ML cell displacement over time in Control, **(B)** *sumf1* OE, **(C)** *sumf2* OE, **(D)** *sumf1+sumf2* OE, **(E)** *sulf1* OE, **(F)** TUwt, **(G)** MZ*sumf1-/-,* **(H)** ABwt, **(I)** MZ*sumf2-/-,* **(J)** MZ*sumf1-/-;* MZ*sumf2-/-, and* (**K**) MZ*sulf1-/-* embryos. Each line represents mean and standard error for measured cells within a single embryo. Dotted line shows typical onset of convergence movements in WT control embryos around 8 hpf. Source data are provided as a supplementary Source Data file.

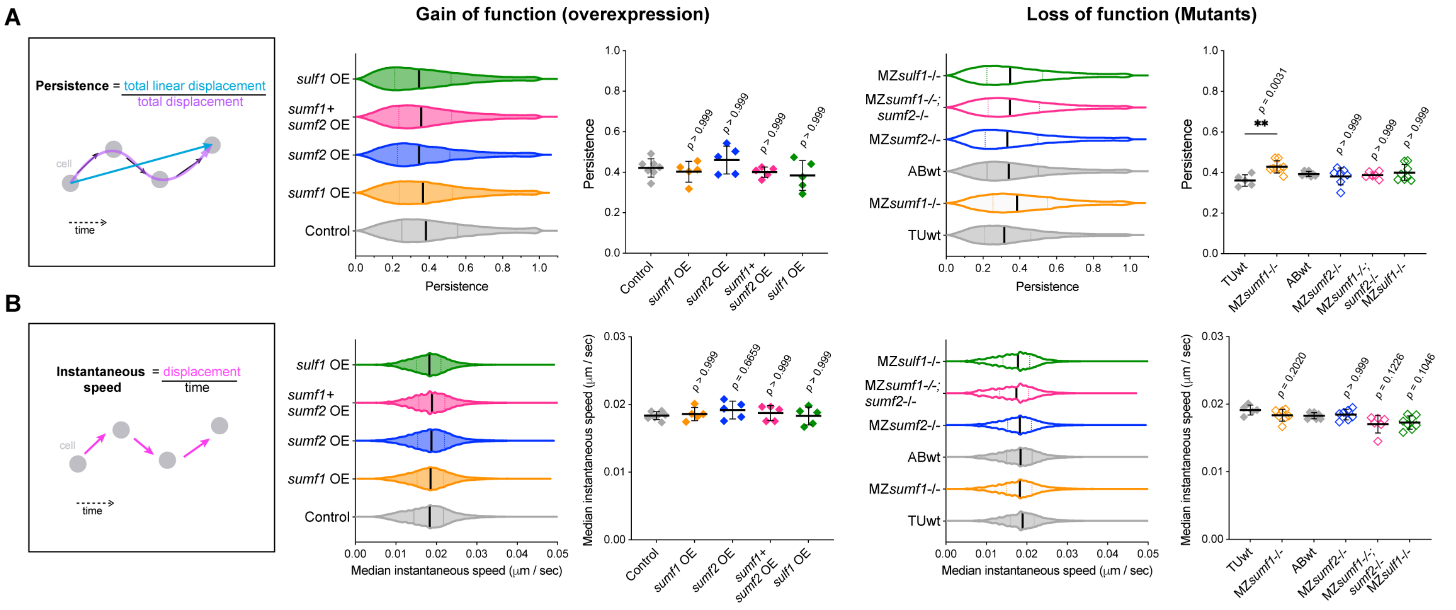

Supplementary Fig. 7. Altering *sumf1, sumf2,* and *sulf1* levels does not broadly impact cellular motion during C&E. (A) Individual cell persistence measurements were calculated as the ratio between the linear distance traveled by the cell and the total length of its path. **(B)** Individual cell instantaneous speed was calculated as the median distance traveled by a cell over time between consecutive time frames (5 minutes). Violin plots include measured cells from all embryos of a given condition combined; black solid lines and dotted lines indicate the median and the first and third quartiles, respectively. Scatter plots represent embryo-level averages; each point represent a single embryo, means and standard deviation are indicated. * p<0.05, ** p<0.01, Mann-Whitney test. Source data are provided as a supplementary Source Data file.

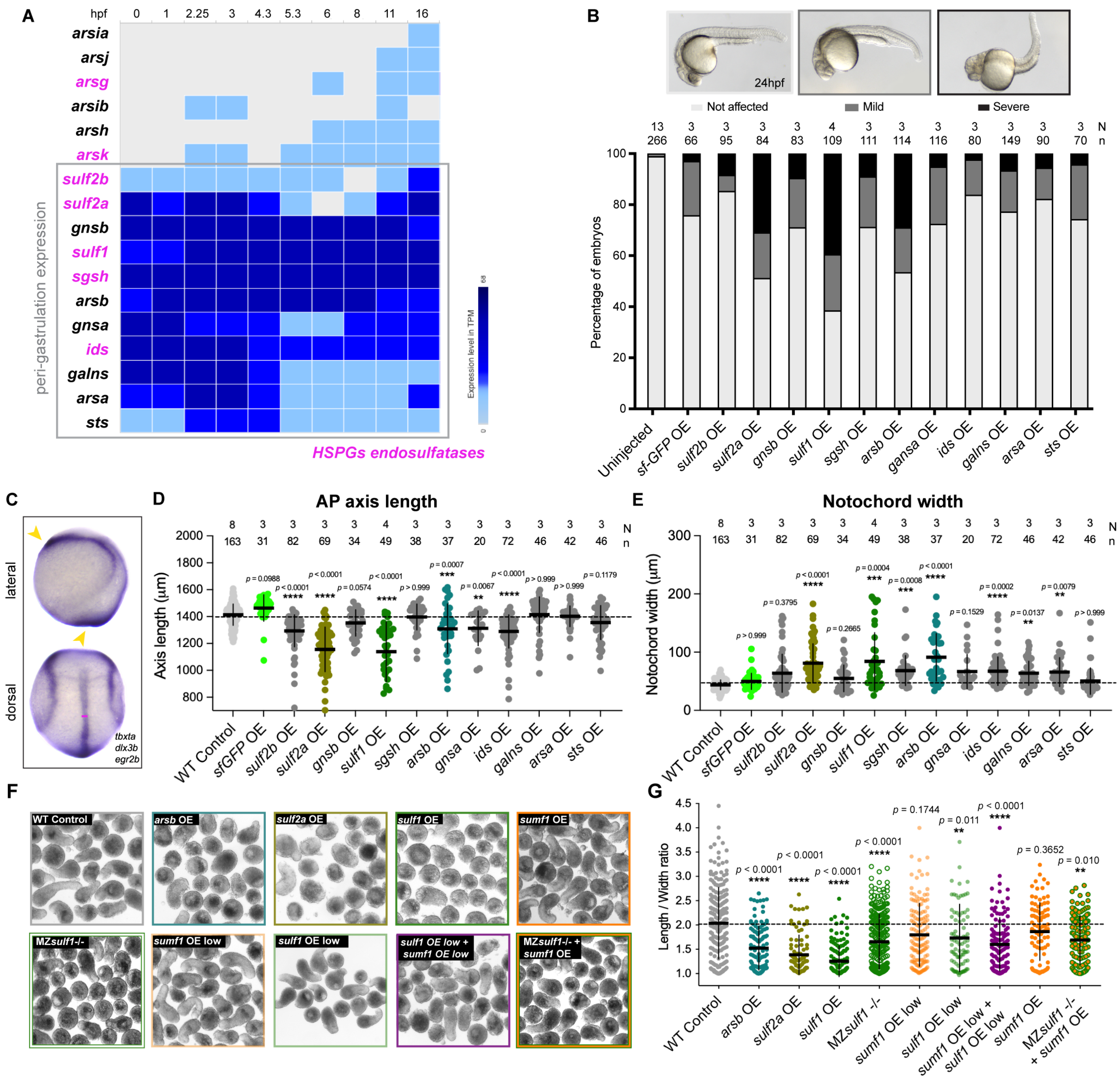

Supplementary Fig. 8. Evaluation of C&E defects upon sulfatase overexpression. (A) Expression levels of all 17 zebrafish sulfatases over time based on publicly available bulk RNA-seq data. Gray square indicates the 11 expressed during peri-gastrulation, HSPG endosulfatases are in magenta. **(B)** (Top) Representative images of 24 hpf embryos overexpressing each of the 11 peri-gastrulation sulfatases displaying a range of axis phenotypes scored as *"Not affected"*, *"Mild"* or *"Severe"*. (Bottom) Mean percentage of 24 hpf embryos of the conditions indicated exhibiting each class of axis defect. N: number of independent experiments, n: number of embryos. **(C)** WISH for *tbxta* (notochord), *dlx3b* (neural border) and *egr2b* (rhombomeres 3 & 5) in tailbud stage (10 hpf) embryos shown from lateral (top) and dorsal (bottom) views. **(D, E)** Anteroposterior (AP) axis length (D) and notochord width (E) (as in Figure 2) in control and sulfatase-overexpressing embryos. Each dot represents a single embryo. Means and standard deviation are indicated. (** p<0.01, *** p<0.001, **** p<0.0001 as compared with WT control group by Kruskal–Wallis and Dunn's multiple comparisons tests. **(F)** Representative images of *acvr1b** explants from the indicated experimental conditions. **(G)** Length/width ratios of 12 hpf explants shown in (F). Each dot represents a single explant. Means and standard deviation are indicated. * p>0.05, ** p<0.01, **** p<0.0001 compared with WT control group by Kruskal–Wallis and Dunn's multiple comparisons tests. Source data are provided as a supplementary Source Data file.

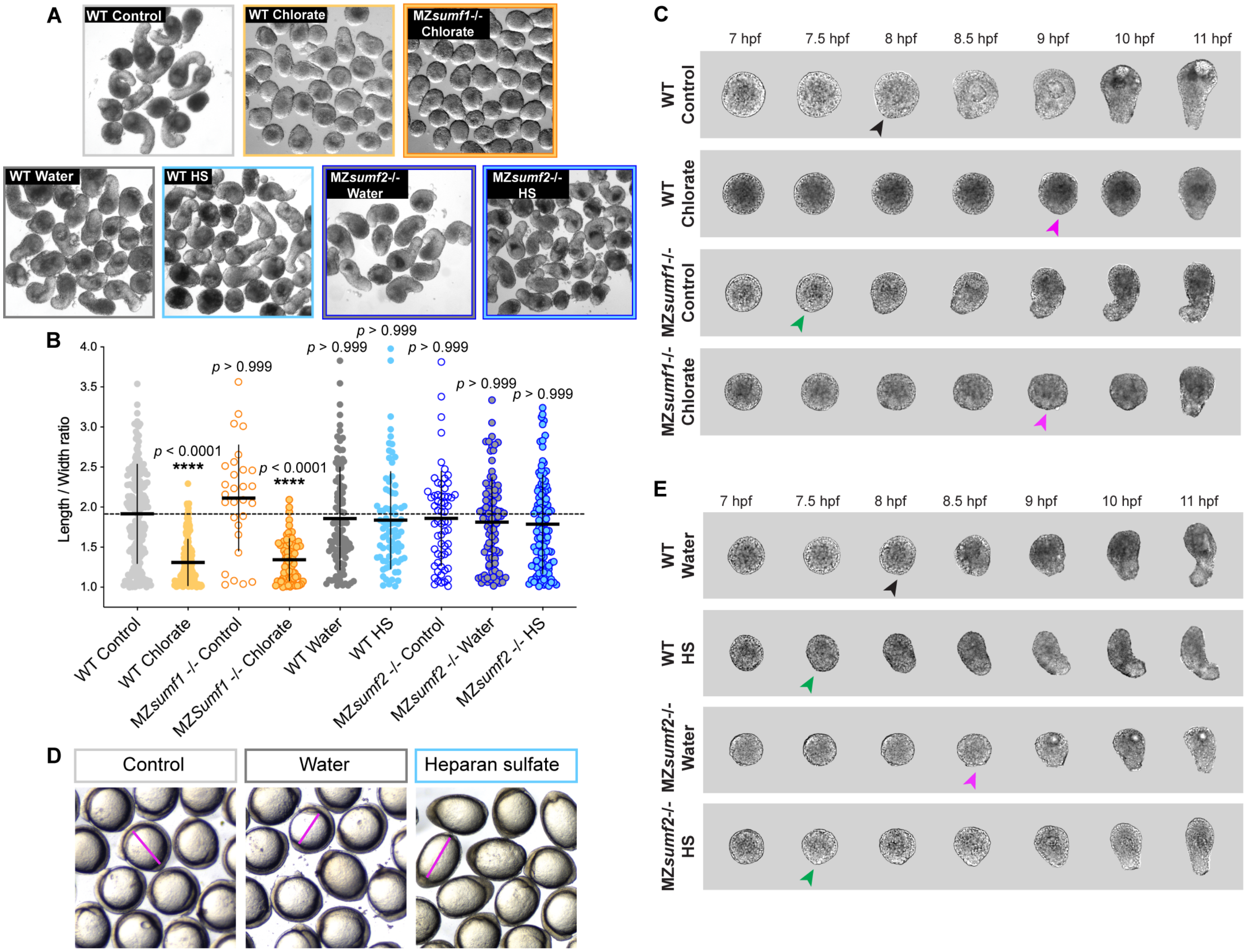

Supplementary Fig. 9. Effect of reduced sulfation and increased heparan sulfate on C&E *ex vivo*. **(A)** Representative images of *acvr1b** explants of the indicated conditions at 12 hpf. **(B)** Length/width ratios of explants shown in (A). Each dot represents a single explant. Means and standard deviation are indicated. **** $p<0.0001$ compared to WT controls by Kruskal–Wallis and Dunn's multiple comparisons tests. **(C)** Representative bright-field images of *acvr1b** explants of the indicated genotypes and treatments over time. Black, magenta, and green arrowheads indicate timely, precocious, and delayed onset of extension, respectively. **(D)** Heparan sulfate (HS) injected embryos display a dorsalized phenotype at the 2-somite stage, indicated by increased animal–vegetal length (magenta lines). **(E)** Representative bright-field images of *acvr1b** explants of the indicated genotypes and treatments over time, as in (C). Source data are provided as a supplementary Source Data file.
