# Supplementary material for: Sulfatase modifying factors control the timing of zebrafish convergence and extension morphogenesis
Source: Nat Commun. 2026 Mar 31;17:4632. doi: 10.1038/s41467-026-70804-6 (PMC13199485; doi:10.1038/s41467-026-70804-6)
Supplement: Supplementary file 3 — Description of Additional Supplementary Files [file 41467_2026_70804_MOESM3_ESM.pdf]

Supplementary Data 1. Longitudinal RNA-sequencing analysis of zebrafish explants and intact embryos to identify candidate "trigger" genes. (A) RNA-sequencing was performed on zebrafish explants under three conditions (uninjected, *ndr2*, and *acvr1b**) collected at seven developmental stages, corresponding to their intact sibling embryos: sphere, 30% epiboly, 50% epiboly, shield, 75% epiboly, 90% epiboly, and 2-somite stage *(71).* We identified 180 genes whose expression increased at (but not before) shield stage (6 hpf) relative to the sphere stage (4 hpf) in all three conditions, expressed as $\log_2$ fold change. **(B)** The expression profiles of these 180 genes were then compared with RNA-sequencing data from intact embryos at equivalent stages (expressed in TPM) *(74)*. Of these, 129 genes increased between 50% epiboly and shield stages *in vivo*. From this group, we selected genes exhibiting at least a 50% increase between 50% epiboly and shield stages and greater than 5 transcripts per million (TPM) at shield stage in intact embryos. This analysis yielded a list of 28 candidate "trigger" genes, highlighted in yellow. **(C)** Functional annotation of the 28 candidate genes shown in (B).

Supplementary Movie 1. *acvr1b explant extension upon *sumf1* and *sumf2* OE or deficiency.** Representative time-lapse recordings of *acvr1b** explants of the indicated genotypes and conditions undergoing extension. Black, red, and green asterisks indicate timely, precocious, and delayed onset of extension, respectively.

Supplementary Movie 2. Convergence movements in zebrafish gastrulae upon *sumf1* and *sumf2* OE or deficiency. Representative time-lapse series of automated nuclear tracking in the dorsal hemisphere of zebrafish gastrulae of the indicated genotypes and conditions, starting before C&E onset (7 hpf). Tracks are color-coded by mean speed (as in Fig. 3).

Supplementary Movie 3. *acvr1b explant extension upon *sulf1* OE or deficiency.** Representative time-lapse recordings of *acvr1b** explants of the indicated genotypes and conditions undergoing extension. Black, red, and green asterisks indicate timely, precocious, and delayed onset of extension, respectively.

Supplementary Movie 4. Convergence movements in zebrafish gastrulae upon *sulf1* OE or deficiency. Representative time-lapse series of automated nuclear tracking in the dorsal hemisphere of zebrafish gastrulae of the indicated genotypes and conditions, starting before C&E onset (7 hpf). Tracks are color-coded by mean speed (as in Fig. 3).
