# Supplementary material for: Sulfatase modifying factors control the timing of zebrafish convergence and extension morphogenesis
Source: Nat Commun. 2026 Mar 31;17:4632. doi: 10.1038/s41467-026-70804-6 (PMC13199485; doi:10.1038/s41467-026-70804-6)
Supplement: Supplementary file 9 — Reporting Summary [file 41467_2026_70804_MOESM9_ESM.pdf]

nature portfolio

Corresponding author(s): Margot Kossmann Williams

Last updated by author(s): Mar 2, 2026

Reporting Summary

Nature Portfolio wishes to improve the reproducibility of the work that we publish. This form provides structure for consistency and transparency in reporting. For further information on Nature Portfolio policies, see our Editorial Policies and the Editorial Policy Checklist.

Statistics

For all statistical analyses, confirm that the following items are present in the figure legend, table legend, main text, or Methods section.

n/a	Confirmed	
☐	☒	The exact sample size (*n*) for each experimental group/condition, given as a discrete number and unit of measurement
☐	☒	A statement on whether measurements were taken from distinct samples or whether the same sample was measured repeatedly
☐	☒	The statistical test(s) used AND whether they are one- or two-sided<br>*Only common tests should be described solely by name; describe more complex techniques in the Methods section.*
☐	☒	A description of all covariates tested
☐	☒	A description of any assumptions or corrections, such as tests of normality and adjustment for multiple comparisons
☐	☒	A full description of the statistical parameters including central tendency (e.g. means) or other basic estimates (e.g. regression coefficient) AND variation (e.g. standard deviation) or associated estimates of uncertainty (e.g. confidence intervals)
☐	☒	For null hypothesis testing, the test statistic (e.g. F, t, r) with confidence intervals, effect sizes, degrees of freedom and P value noted<br>*Give P values as exact values whenever suitable.*
☒	☐	For Bayesian analysis, information on the choice of priors and Markov chain Monte Carlo settings
☒	☐	For hierarchical and complex designs, identification of the appropriate level for tests and full reporting of outcomes
☒	☐	Estimates of effect sizes (e.g. Cohen's *d*, Pearson's *r*), indicating how they were calculated

Our web collection on statistics for biologists contains articles on many of the points above.

Software and code

Policy information about availability of computer code

Data collection	Microscopy data were collected using Nikon NIS Elements software.
Data analysis	GraphPad Prism 10, ImageJ/FIJI (using standard shape descriptors and the TrackMate7 plugin), Jalview2.11.5.1 and CellPose 2.0 implemented in Python were used for data analysis.

For manuscripts utilizing custom algorithms or software that are central to the research but not yet described in published literature, software must be made available to editors and reviewers. We strongly encourage code deposition in a community repository (e.g. GitHub). See the Nature Portfolio guidelines for submitting code & software for further information.

Data

Policy information about availability of data

All manuscripts must include a data availability statement. This statement should provide the following information, where applicable:

- Accession codes, unique identifiers, or web links for publicly available datasets
- A description of any restrictions on data availability
- For clinical datasets or third party data, please ensure that the statement adheres to our policy

Raw RNA-sequencing data were previously published and are available in NCBI Gene Expression Omnibus under accession number GSE246158 [https://www.ncbi.nlm.nih.gov/geo/query/acc.cgi?acc=GSE246158]. Raw data for LC-MS analysis of glycosaminoglycans generated in this study are available at GlycoPOST under project ID GPST000676 [http://doi.org/10.50821/GLYCOPOST-GPST000676]. All image and gene expression analysis data generated for this study are available in the Supplementary and Source Data files. Source data are provided with this paper.

Research involving human participants, their data, or biological material

Policy information about studies with human participants or human data. See also policy information about sex, gender (identity/presentation), and sexual orientation and race, ethnicity and racism.

Reporting on sex and gender	*Use the terms sex (biological attribute) and gender (shaped by social and cultural circumstances) carefully in order to avoid confusing both terms. Indicate if findings apply to only one sex or gender; describe whether sex and gender were considered in study design; whether sex and/or gender was determined based on self-reporting or assigned and methods used.*<br>*Provide in the source data disaggregated sex and gender data, where this information has been collected, and if consent has been obtained for sharing of individual-level data; provide overall numbers in this Reporting Summary. Please state if this information has not been collected.*<br>*Report sex- and gender-based analyses where performed, justify reasons for lack of sex- and gender-based analysis.*
Reporting on race, ethnicity, or other socially relevant groupings	*Please specify the socially constructed or socially relevant categorization variable(s) used in your manuscript and explain why they were used. Please note that such variables should not be used as proxies for other socially constructed/relevant variables (for example, race or ethnicity should not be used as a proxy for socioeconomic status).*<br>*Provide clear definitions of the relevant terms used, how they were provided (by the participants/respondents, the researchers, or third parties), and the method(s) used to classify people into the different categories (e.g. self-report, census or administrative data, social media data, etc.)*<br>*Please provide details about how you controlled for confounding variables in your analyses.*
Population characteristics	*Describe the covariate-relevant population characteristics of the human research participants (e.g. age, genotypic information, past and current diagnosis and treatment categories). If you filled out the behavioural & social sciences study design questions and have nothing to add here, write "See above."*
Recruitment	*Describe how participants were recruited. Outline any potential self-selection bias or other biases that may be present and how these are likely to impact results.*
Ethics oversight	*Identify the organization(s) that approved the study protocol.*

Note that full information on the approval of the study protocol must also be provided in the manuscript.

Field-specific reporting

Please select the one below that is the best fit for your research. If you are not sure, read the appropriate sections before making your selection.

☒ Life sciences ☐ Behavioural & social sciences ☐ Ecological, evolutionary & environmental sciences

For a reference copy of the document with all sections, see nature.com/documents/nr-reporting-summary-flat.pdf

Life sciences study design

All studies must disclose on these points even when the disclosure is negative.

Sample size	While no formal power calculation was performed, sample sizes of ≥30 explants, ≥15 fixed embryos, or ≥5 live embryos per condition were chosen based on prior experience and standards in the field, providing adequate statistical power to detect biologically relevant differences using the statistical tests employed.
Data exclusions	No data were excluded from the analyses in this study.
Replication	All experiments were performed in at least three independent replicates. All attempts at replication were successful.
Randomization	Fish were chosen from their home tank to be crossed at random, and embryos were randomly chosen from each clutch for injection and inclusion in experiments.
Blinding	Researchers were kept unaware of the conditions of all image data using the blind_renamer Perl script (https://github.com/jimsalterjrs/blindanalysis) (blindanalysis: v.1.0.) prior to analysis. This program renames images with a random string of characters and generates a key file which was used to un-blind images only after analysis.

Reporting for specific materials, systems and methods

We require information from authors about some types of materials, experimental systems and methods used in many studies. Here, indicate whether each material, system or method listed is relevant to your study. If you are not sure if a list item applies to your research, read the appropriate section before selecting a response.

Materials & experimental systems

n/a	Involved in the study	
☐	☒	Antibodies
☒	☐	Eukaryotic cell lines
☒	☐	Palaeontology and archaeology
☐	☒	Animals and other organisms
☒	☐	Clinical data
☒	☐	Dual use research of concern
☒	☐	Plants

Methods

n/a	Involved in the study	
☒	☐	ChIP-seq
☒	☐	Flow cytometry
☒	☐	MRI-based neuroimaging

Antibodies

Antibodies used

Anti-digoxigenin (Roche #11093274910)

Validation

This antibody has been used for decades by hundreds of zebrafish researchers to perform whole mount in situ hybridization (WISH). When using a new vial of the antibody for the first time, we first test it in a WISH for tbxta, a robustly expressed developmental gene with a well described expression pattern, to ensure the antibody produces the expected staining pattern.

Animals and other research organisms

Policy information about studies involving animals; ARRIVE guidelines recommended for reporting animal research, and Sex and Gender in Research

Laboratory animals

Adult zebrafish (Danio rerio) of the AB and TU strains (obtained from the zebrafish international resource center (ZIRC) via the Baylor College of Medicine zebrafish facility) were used to obtain WT embryos for controls and overexpression experiments. Mutant lines used include sumf1 la015919Tg (obtained from ZIRC), sulf1 sjr9 (obtained from the University of Montreal), sumf2 bcm126 (generated for this study at Baylor College of Medicine), and oep tz257 (obtained from Washington University School of Medicine). Experimental procedures and analysis were performed from the one-cell stage to 48 hours post fertilization.

Wild animals

This study did not involve wild animals.

Reporting on sex

Sex was not considered a relevant variable in this study because sex is not determined in zebrafish embryos until approximately 2 weeks post fertilization, well after the period of early embryogenesis examined in this study.

Field-collected samples

This study did not involve field-collected samples.

Ethics oversight

This study was conducted in in compliance with the Baylor College of Medicine Institutional Animal Care and Use Committee, Animal Welfare Assurance number D16-00475, approved protocol number AN-8127. Work involving recombinant nucleic acids was approved by the Baylor College of Medicine Institutional Biosafety committee, approved protocol number D-633.

Note that full information on the approval of the study protocol must also be provided in the manuscript.

Plants

Seed stocks

Report on the source of all seed stocks or other plant material used. If applicable, state the seed stock centre and catalogue number. If plant specimens were collected from the field, describe the collection location, date and sampling procedures.

Novel plant genotypes

Describe the methods by which all novel plant genotypes were produced. This includes those generated by transgenic approaches, gene editing, chemical/radiation-based mutagenesis and hybridization. For transgenic lines, describe the transformation method, the number of independent lines analyzed and the generation upon which experiments were performed. For gene-edited lines, describe the editor used, the endogenous sequence targeted for editing, the targeting guide RNA sequence (if applicable) and how the editor was applied.

Authentication

Describe any authentication procedures for each seed stock used or novel genotype generated. Describe any experiments used to assess the effect of a mutation and, where applicable, how potential secondary effects (e.g. second site T-DNA insertions, mosiacism, off-target gene editing) were examined.
